# Spectrophotometric Determination of Phenolic Antioxidants in the Presence of Thiols and Proteins

**DOI:** 10.3390/ijms17081325

**Published:** 2016-08-12

**Authors:** Aslı Neslihan Avan, Sema Demirci Çekiç, Seda Uzunboy, Reşat Apak

**Affiliations:** 1 Department of Chemistry, Faculty of Engineering, Istanbul University, 34320 Istanbul, Turkey; neslihanavan@hotmail.com (A.N.A.); sema@istanbul.edu.tr (S.D.Ç.); sedauzunboy@hotmail.com (S.U.); 2 Turkish Academy of Sciences (TUBA) Piyade St. No. 27, 06690 Çankaya Ankara, Turkey

**Keywords:** antioxidant capacity assays, CUPRAC, ABTS, FRAP, thiol stabilization, Hg(II)-thiol reaction

## Abstract

Development of easy, practical, and low-cost spectrophotometric methods is required for the selective determination of phenolic antioxidants in the presence of other similar substances. As electron transfer (ET)-based total antioxidant capacity (TAC) assays generally measure the reducing ability of antioxidant compounds, thiols and phenols cannot be differentiated since they are both responsive to the probe reagent. In this study, three of the most common TAC determination methods, namely cupric ion reducing antioxidant capacity (CUPRAC), 2,2′-azinobis(3-ethylbenzothiazoline-6-sulfonic acid) diammonium salt/trolox equivalent antioxidant capacity (ABTS/TEAC), and ferric reducing antioxidant power (FRAP), were tested for the assay of phenolics in the presence of selected thiol and protein compounds. Although the FRAP method is almost non-responsive to thiol compounds individually, surprising overoxidations with large positive deviations from additivity were observed when using this method for (phenols + thiols) mixtures. Among the tested TAC methods, CUPRAC gave the most additive results for all studied (phenol + thiol) and (phenol + protein) mixtures with minimal relative error. As ABTS/TEAC and FRAP methods gave small and large deviations, respectively, from additivity of absorbances arising from these components in mixtures, mercury(II) compounds were added to stabilize the thiol components in the form of Hg(II)-thiol complexes so as to enable selective spectrophotometric determination of phenolic components. This error compensation was most efficient for the FRAP method in testing (thiols + phenols) mixtures.

## 1. Introduction

There is a critical balance between reactive oxygen/nitrogen species (ROS/RNS) and antioxidants (AOx) in the human body. Although there are some important endogenous AOx sources, such as small molecule antioxidants (bilirubin, uric acid, glutathione, etc.) and enzymes (e.g., catalase, superoxide dismutase and glutathione reductase), substantial amounts of AOx are taken by foods. Polyphenols have a special importance among dietary antioxidants as being the most consumed phytochemicals. Plenty of foodstuffs and beverages can be mentioned as dietary polyphenol sources such as vegetables, fruits, juices, tea and coffee. Polyphenol intake is generally accepted to be effective on the prevention of oxidative stress-originated diseases, such as cardiovascular and neurodegenerative diseases and cancer. Polyphenols are usually accepted as antioxidants serving cell survival and contributing to the regulation of cellular redox status; they may interfere at the initiation, development, and progression of cancer through the modulation of certain cellular processes and signaling pathways [1]. However, they may also act as pro-oxidants under certain circumstances and help the prevention of tumor growth [2].

An antioxidant can be defined as a substance (small molecule or complex system) that, when added to an oxidizable molecule in small amounts, is able to protect such molecules by delaying, retarding, or inhibiting their autoxidation (where the protected substrate is usually a biomacromolecule, like a lipid, protein, or DNA) [3]. Though used interchangeably, “antioxidant activity” and “antioxidant capacity” terms are not identical, as the former deals with the rate, while the latter is concerned with the thermodynamic conversion of antioxidant action. Foti argues that in order to establish whether a test compound (AH) is a potent antioxidant or not, it is necessary to compare the rate at which AH quenches peroxyl radicals to the rate at which peroxyl radicals attack the substrate [4]. Valgimigli and Amorati are of the opinion that certain antioxidant activity measurement methods have different soundness and are often used with little consideration of the chemistry behind them, and that some yield numerical values or rankings of antioxidant performance not corresponding to physical/biochemical reality [5].

Total antioxidant capacity (TAC) is a useful parameter reflecting the cumulative effect of several antioxidants in complex matrices rather than individual antioxidative properties of relevant components. By definition, TAC should be equal to the sum of the antioxidant capacities of components constituting a mixture. TAC is usually measured by several spectrophotometric tests, but absorbances of mixture components is not always additive, and synergistic or antagonistic effects may frequently emerge as a result of interactions among several components. These different interactions can be explained by AOx structure and reactivity. Knowing the initial antioxidant capacity of a single component can give useful information to solve this interaction problem [6]. Wang et al. concluded that antioxidant interactions can not only result in positive effects but also could produce negative effects on the total antioxidant capacities of foods or diets [7].

It is natural that in vitro investigation of the antioxidant activity of a given antioxidant compound cannot provide enough knowledge about its bioavailability. Due to the complexity of foodstuffs, combinations of antioxidants may cause a great variety of interactions among them (such as “synergistic”, where the whole exceeds the sum, or “antagonistic”, whereas the whole stays behind the sum of individual antioxidative powers) and in vivo activities of bioavailability and metabolism may add more complex interactions to the existing ones. In a specific study on berry fruits, researchers reported that the TAC of suitable combinations of the tested samples were higher than the sum of the corresponding individual antioxidant capacities [8]. In another study, Wang et al. prepared different combinations of three fruits, four vegetables, and four legumes and they investigated the TAC values of these mixtures using ferric reducing antioxidant power (FRAP), 2,2-diphenyl-1-picryl-hydrazyl (DPPH^•^), and oxygen radical absorbance capacity (ORAC) methods. The authors reported some synergistic and antagonistic interactions, and more than half of these combinations gave additive results. Among the tested samples, mixtures of raspberry and adzuki bean extracts showed synergistic interactions in all three TAC determination methods [7]. In addition, Iacopini et al. investigated catechin, epicatechin, quercetin, rutin, and resveratrol in red grape using high performance liquid chromatography–ultra violet detector (HPLC–UV). They also investigated the reactive species scavenging ability of these compounds using DPPH and peroxynitrite (ONOO^−^). The researchers reported a potential for synergistic interaction towards ONOO^−^ consumption for quercetin, rutin, and resveratrol combinations. On the other hand, they reported an additive effect between catechin and epicatechin [9]. In addition to in vitro TAC, some researchers were interested in in vivo effects of polyphenolic antioxidants. In a critical review, Fraga investigated the antioxidant action mechanism of these compounds under in vivo conditions with a thorough evaluation of free radical scavenging and metal chelating effects added to some protein and lipid interactions [10]. Although the exact mechanism of synergistic action of antioxidants is unknown, it may be speculated for lipid peroxidation and membrane protection that synergism arises when one antioxidant may spare or regenerate another during the course of oxidation (e.g., as seen in ascorbic acid and α-tocopherol pair, via ascorbate reduction of oxidized vitamin E back to the original α-tocopherol in vivo) [11].

Interactions between polyphenols and proteins have been highly investigated. Gilani et al. reported a non-covalent binding between polyphenols and proteins [12]. It is known that astringency in the polyphenol-rich foods, such as tea, is the consequence of interactions of certain polyphenols and salivary proteins [13]. Plant-derived food phenolic compounds interact with saliva proteins, presenting a great variety; especially, proline-rich proteins can bind phenolic compounds [14]. It is also known that resveratrol has weak water solubility, and to obtain a certain concentration, it should be bound to plasma proteins, such as human serum albumin (HSA) and hemoglobin (Hb) [15]. Dufor and Dangles reported flavonoid-HSA complexation in their study [16]. There are also different studies on interactions of flavonoids and food proteins such as ovalbumin, gelatin, α-lactalbumin, and milk proteins (β-casein, β-lactoglobulin); amino acid composition and protein conformation are the two important factors affecting these interactions [17]. Polyphenol-protein interactions can be reversible or irreversible. Irreversible interactions are usually a consequence of covalent bonding, whereas in reversible interactions, polyphenols and proteins are held together with non-covalent forces (hydrogen bonding, van der Waals forces, etc.) [18]. These interactions may affect the measured TAC. Arts et al. noted that the trolox equivalent antioxidant capacity (TEAC) antioxidant capacity of several components of green and black tea with α-, β-, and κ-casein or albumin was not additive, and that a part of the total antioxidant capacity was masked by protein-flavonoid interactions. They also observed a maximum masking effect in the presence of β-casein, epigallocatechin gallate, and gallate combinations, so they reported that the efficiency of antioxidants depends on sample matrix [19]. In another study, Lorenz et al. expressed an antagonistic interaction between tea polyphenols and milk proteins. As a result of these interactions, the authors concluded that consumption of tea with milk may reduce the beneficial effect of tea on vascular diseases, and that increasing milk content of epicatechin-containing chocolate may decrease its health assistance [20]. Likewise, Gallo et al. concluded with the help of mass spectrometry and antioxidant activity measurements that various milk protein fractions caused a decrease in the antioxidant activity of cocoa polyphenols [21].

Interactions of small molecule thiols with polyphenols were also investigated. Fujimoto and Masuda examined the interactions between polyphenols and thiols under radical oxidation conditions and studied resulting cross-coupling products using liquid chromatography–mass spectrometry (LC–MS) [22]. Boots et al. identified adducts between oxidized quercetin and glutathione (GSH), and reported that, in the absence of GSH, oxidized quercetin could give harm to some vital enzymes [23]. Awad et al. also investigated the formation of reversible glutathionyl flavonoid adducts [24].

Some antioxidant researchers argue that in physiologically-relevant antioxidant activity testing, there should be an oxidizable substrate (i.e., lipid, protein, DNA) whose oxidation inhibition by the antioxidant is to be measured. Direct (competitive) antioxidant assays involve a fluorogenic or chromogenic probe and biologically-relevant reactive species (i.e., ROS/RNS), whereas in indirect (non-competitive) antioxidant assays, physiological redox reactions are simulated on an artificial probe without a biologically-relevant reactive species [25]. In this regard, electron transfer-based antioxidant capacity assays like trolox equivalent antioxidant capacity (ABTS/TEAC), FRAP, and cupric ion reducing antioxidant capacity (CUPRAC) are non-competitive, using a single probe which changes or loses color upon reduction by antioxidants. Naturally, non-competitive TAC assays do not necessarily yield the same antioxidant ranking as physiologically-relevant antioxidant activity tests (e.g., inhibition of lipid peroxidation). For example, the CUPRAC test gives an antioxidant capacity order for hydroxy-cinnamic acids as caffeic > ferulic > *p*-coumaric acids in accordance with the inhibitive order of low density lipoprotein (LDL) oxidation, whereas Rice-Evans et al. gives the reverse order for the ABTS/TEAC assay (i.e., *p*-coumaric > ferulic > caffeic acids) [26,27,28].

It would be interesting to see whether covalent or non-covalent bindings of thiols to polyphenols would indeed give rise to significant deviations from additivity of absorbances in corresponding mixtures. In the presented study, three of the most common TAC determination methods, namely FRAP [29], ABTS/TEAC [30], and CUPRAC [31], were applied to different phenolic AOx in the presence of selected thiols and proteins. Since precise additivity was observed in the mentioned mixtures only by using the CUPRAC method, mercury(II) salts were added to stabilize thiols in such mixtures in order to compensate for deviations noted with FRAP and ABTS/TEAC methods. In accordance with the Hard and Soft Acids and Bases (HSAB) Theory [32], Hg(II) is characterized as Class B (soft Lewis acid) metal ion with highly-polarizable outer shell electrons, preferring soft Lewis base sulfur-donor ligands to form stable complexes. It is a well-known phenomenon that mercury tends to bind sulfur-containing proteins and GSH [33].

## 2. Results

### 2.1. Optimization of the Amount of Hg(Ac)_2_ for the Ferric Reducing Antioxidant Power (FRAP) Method

The FRAP reagent, i.e., Fe(III)-2,4,6-tris(2-pyridyl)-*S*-triazine (TPTZ) complex, accepts an electron from antioxidants to form the chromophore (Fe(II)-TPTZ chelate), and the absorbance increase at 595 nm is related to AOx concentration. The FRAP method was applied as stated in Section 4.6. for gallic acid (GA) (alone), gallic acid + cysteine (GA + CYS), and GA + CYS + Hg^2+^ mixture solutions. For a series of CYS:Hg^2+^ solutions prepared at 1:0.5, 1:1, 1:2.5, 1:5, and 1:10 mol/mol ratios, the measured absorbances (with roughly ±5% deviation) are shown in Table 1.

As can be seen from Table 1, although CYS alone was nearly non-responsive to FRAP, the FRAP absorbance (A_FRAP_) of (CYS + GA) greatly exceeded the sum of the absorbances of CYS and GA. This obvious synergetic effect could be overcome by the addition of Hg(II) acting as a selective complexing agent for thiol, which resulted in the restoration of the individual absorbance of GA in the mixture (Table 1). For Hg(II):CYS mole ratios ≥1:1, Hg(II) was effective in thiol stabilization of mixtures and one could obtain only GA absorbance. However, when Hg(II):CYS ratio exceeded 2.5, a slight turbidity was observed which may apparently increase the absorbance. Therefore, this ratio was set at an optimal value of 2.5 for further experiments.

### 2.2. FRAP Method Experiments

For selected thiol compounds (homocysteine (HCYS), *N*-acetyl-l-cysteine (NAC)), phenolic AOxs (gallic acid (GA), caffeic acid (CFA), catechin (CAT), epicatechin (EC)), and different (thiol + polyphenol) binary mixtures, the FRAP method was applied (as stated in Section 4.6.) in the presence and absence of Hg(Ac)_2_. To calculate percentage relative error, RE% = |[(A_exp_ − A_theo_)/A_theo_]| × 100 formula was used (A_exp_: experimentally found absorbance, A_theo_: theoretically calculated absorbance, as the mathematical sum of individual absorbance values for the binary mixtures). The results are shown in Table 2.

As can be seen from Table 2, the presence of thiol compounds caused a huge difference in the absorbance of (thiol + phenolic) mixtures and, consequently, very high RE% values. In the presence of Hg^2+^ salts, the experimental absorbance values for binary mixtures were very close to the theoretical ones, and the resulting RE values were roughly between 1% and 9%.

**Figure 1:**
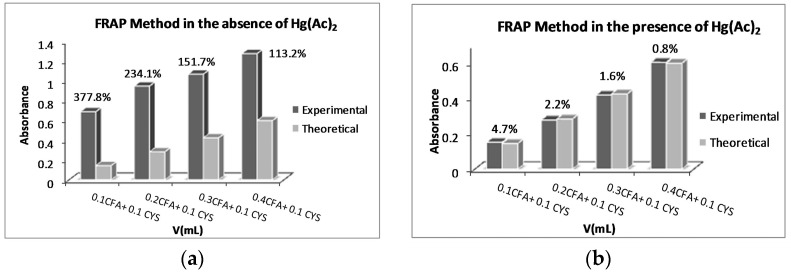
Binary mixtures of 0.1 mL of 2.0 × 10^−4^ M cysteine (CYS) and 0.1–0.4 mL volumes of 1.0 × 10^−4^ M caffeic acid (CFA) (**a**) in the absence, and (**b**) presence, of 0.1 mL of 5.0 × 10^−4^ M Hg(Ac)_2_. FRAP: ferric reducing antioxidant power.

Similar binary mixtures were tested for all mentioned polyphenols. The biggest RE (378%) was calculated for the (0.1 mL CFA + 0.1 mL CYS) mixture. On the other hand, when the experiments were repeated in the presence of Hg^2+^, all RE values were reduced to <5%. The absorbance differences in the absence and presence of Hg^2+^ salts are shown in Figure 1.

### 2.3. Optimization of the Amount of Hg(Ac)_2_ for the ABTS/TEAC Method

Hg(II) optimization experiments were performed as stated in Section 4.7., and the obtained results are depicted in Table 3.

As can be seen from Table 3, the presence of Hg(II) as a thiol stabilization agent did not interfere with the ABTS^•+^ reference reading, and for the Hg(II):CYS mol ratio ≥5, the obtained absorbances were nearly the same—within experimental error—as the reference absorbance. We can conclude that Hg(II) could complex all CYS in solution and prevent the oxidation of thiols with ABTS^•+^ (which would normally cause an absorbance drop with respect to that of the ref.). For further experiments, 1:10 mol ratio was selected as optimal.

### 2.4. Measurements with the ABTS/TEAC Method

**Figure 2:**
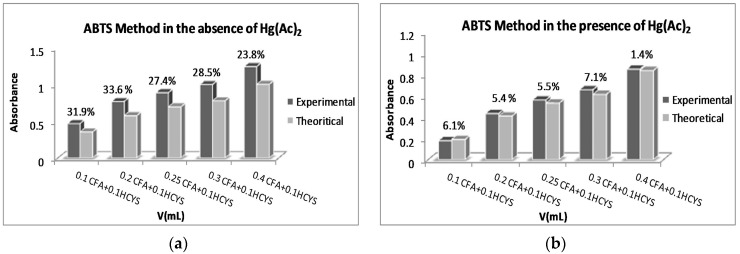
Binary mixtures of 0.1 mL of 1.0 × 10^−4^ M homocysteine (HCYS) and 0.1–0.4 mL volumes of 2.0 × 10^−4^ M CFA (**a**) in the absence, and (**b**) in the presence, of 0.1 mL of 1.0 × 10^−3^ M Hg(Ac)_2_. ABTS: 2,2′-azinobis(3-ethylbenzothiazoline-6-sulfonic acid) diammonium salt.

The ABTS^•+^ chromogenic radical accepts an electron from antioxidants to convert into the colorless ABTS form, where the absorbance decrease at 734 nm is related to AOx concentration. ABTS/TEAC method studies were realized as described in Section 4.7. Absorbance values for all tested samples and reference (ref.) were read against phosphate-buffered saline (PBS) buffer at 734 nm, and ∆A values were calculated as the difference between A_ref._ and A_sample_ (∆A = A_ref._ − A_sample_). The relative errors were calculated by using the equation: RE% = |[(∆A_exp._ − ∆A_theo_)/∆A_theo_]| × 100. For GSH and CAT mixtures, the RE values were calculated between 0.1% and 5.7% even in the absence of Hg^2+^, so these experiments were not repeated in the presence of Hg^2+^. For binary mixtures of 0.1 mL of 1.0 × 10^−4^ M HCYS (or NAC) and 0.05–0.25 mL volumes of 5.0 × 10^−5^ CAT, the results are depicted in Table 4. For 0.1 mL of 1.0 × 10^−4^ thiol compounds and 0.05–0.25 mL EC mixtures, the lowest RE was calculated as 0.5% for HCYS + 0.1 mL EC and the highest RE was 23% for NAC + 0.1 mL EC mixtures; however, the RE was brought down to 2.4% for the latter mixture in the presence of Hg^2+^. For mixtures consisting of 0.1 mL of 1.0 × 10^−4^ M thiol compound and 2.0 × 10^−4^ M CFA, the lowest RE was calculated as 0.8% for CYS + 0.3 mL CFA and the highest RE was calculated to be 33.6% for HCYS + 0.2 mL of the CFA mixture; however, the RE was brought down to 5.4% in the presence of Hg^2+^. All results are summarized in Figure 2.

As can be seen from Table 4, the presence of Hg^2+^ provided better RE results, i.e., Hg(II) addition brought relative errors up to 22% down to <10%.

For ternary mixtures, the RE values ranged between 1.6%–14.3% for 0.1 mL of 1.0 × 10^−4^ M GSH + 0.1 mL of 4.0 × 10^−5^ M GA + 5.0 × 10^−5^ M CAT and 0.1 mL of 0.1 × 10^−4^ M CYS + 0.1 mL of 2.0 × 10^−4^ M CFA + 0.1 mL of 5.0 × 10^−5^ M EC, respectively. When the same experiments were repeated in the presence of Hg^2+^, the RE for CYS + CFA + EC was calculated as 1.6%, again confirming the thiol-stabilizing effect of added Hg(II).

### 2.5. ABTS/TEAC Method Experiments for Polyphenol–Protein Mixtures

ABTS/TEAC method experiments for polyphenol–protein mixtures were applied as stated in Section 4.9. For all tested combinations of CAT and CFA + casein mixtures, ABTS/TEAC results were additive. When different amounts of CFA and CAT were added to 0.1 mL of 0.125% bovine serum albumin (BSA), the RE values for CFA-BSA mixtures were between 9.2% and 16.0%; and for CAT-BSA mixtures, the RE values were between 8.0% and 19.7%. These experiments were repeated in the presence of 0.1 mL 1.0 × 10^−3^ M Hg(II) but, unfortunately, the results were not better than those in the absence of Hg(II). For CAT and CFA mixtures with 0.3 mL of 1:20 diluted egg white protein solutions, the RE% values were between 3.5 and 12.6 for CAT + egg white protein solution and 0.6–19.3 for CFA + egg white protein solution. The experiments were repeated in the presence of 0.1 mL of 1.0 × 10^−3^ M Hg^2+^, with no significant improvement.

### 2.6. Cupric Ion Reducing Antioxidant Capacity (CUPRAC) Method Experiments for Thiol-Polyphenol Mixtures

The CUPRAC reagent, i.e., Cu(II)-neocuproine complex, accepts an electron from antioxidants to form the orange-yellow colored chromophore (Cu(I)-neocuproine chelate), and the absorbance increase at 450 nm is related to AOx concentration. To investigate the effects of thiols on determination of polyphenols using the CUPRAC method, different binary and ternary thiol-polyphenol mixtures were prepared as stated in Section 4.11. The CUPRAC method was applied to all mentioned compounds (both polyphenols and thiols) individually, and then was applied to binary and ternary mixtures. Theoretical absorbance values were calculated as the mathematical sum of the absorbances due to individual AOx compounds. These values were compared with experimentally-obtained absorbance values of binary and ternary mixtures. Percentage RE values were calculated using the formula |[(A_exp._ − A_theo_)/A_theo_]| × 100. Among all tested TAC methods, CUPRAC gave the most additive results with RE < 5%, without requiring Hg(II) correction. The obtained results for ternary mixtures are shown in Table 5.

### 2.7. CUPRAC Determination of Polyphenols in Mixtures with Hg(Ac)_2_ Correction for Thiols

For CUPRAC measurements of polyphenols in the presence of thiols, different volumes between 0.1 and 0.5 mL of 1.0 × 10^−4^ M CAT were determined in the presence of 0.2 mL of 1.0 × 10^−3^ M GSH or NAC. These mixtures gave perfectly additive results with CUPRAC, and the observed absorbances at 450 nm arose from the Cu(II)-neocuproine oxidation of thiol and polyphenol components in an additive manner. The same experiments were repeated in the presence of 0.1 mL of 1.0 × 10^−2^ M Hg(Ac)_2_ added as a stabilization agent for the thiol component. During the experiments, the thiol compound, phenolic AOx, and Hg^2+^ were mixed together, and this mixture was added as sample into the CUPRAC reagent mixture. After incorporating Hg(II), only the polyphenol component (CAT) responded to the CUPRAC assay, confirming that the polyphenol component of a mixture could be selectively determined with CUPRAC in the presence of a thiol.

### 2.8. CUPRAC Method Experiments for Protein–Polyphenol Mixtures

Related experiments were conducted as mentioned in Section 4.12. For all tested protein–polyphenol mixtures, percentage RE values were lower than 10%, except for CFA-BSA mixtures, where the relative error remained in the range of 10%–17%.

## 3. Discussion

Since polyphenols may exist together with thiol- or protein-type antioxidants in natural and functional food formulations to extend the shelf-life, accurate and precise measurement of the antioxidant capacity of such food mixtures is of vital importance. The definition of TAC usually necessitates the additivity of individual antioxidant capacities of mixture components, but synergistic or antagonistic interactions may occur among various antioxidants giving rise to large deviations from additivity. In this study, three of the most common spectrophotometric TAC determination methods (FRAP, ABTS/TEAC, and CUPRAC) were applied to different thiol-polyphenol and protein-polyphenol mixtures. The most additive results were obtained for the CUPRAC method, and the largest deviations were seen for the FRAP method, whereas ABTS/TEAC exhibited medium level deviations.

The additivity of CUPRAC antioxidant capacities of thiol-containing proteins in admixture with polyphenols was previously shown by the authors’ laboratory [34]. The CUPRAC method has a rather straightforward chemistry toward polyphenols and thiols, oxidizing them to the corresponding quinones and disulfides, respectively, while converting its reagent (cupric-neocuproine) to the CUPRAC chromophore, cuprous-neocuproine complex. Since a single chromophore emerges in the system as a result of the concerned redox reaction, Beer’s law is perfectly obeyed and absorbances are additive for mixtures. However, the ABTS/TEAC method may produce several products from thiols, comprising not only disulfides but sulfinic and sulfonic acids, as well [35]. Thus, perfect additivity may not be expected for thiol-phenol mixtures with the use of the ABTS/TEAC method, and the observed results showed medium level deviations from additivity. On the other hand, even though the FRAP reagent is thermodynamically capable of oxidizing thiols, it cannot do so at an appreciable extent during the protocol time of the FRAP assay, due to kinetic reasons ascribed to the electronic configuration of high-spin Fe(III). Moreover, thiols are usually oxidized through thiyl radicals (RS^•^) [36] which are not formed at an appreciable extent at the acidic pH (i.e., pH 3.6) of the FRAP reaction protocol [25]. As another example of thiyl radical involvement, ferricyanide oxidation of thiols (e.g., 3-mercaptopropionic acid) was demonstrated to generate thiyl radicals, and the pH-dependence of the rate of oxidation suggested that only ionized sulfhydryl groups (RS^−^) were directly involved in the oxidation reaction [37]. Thus, in the presence of polyphenols, thiols may presumably have some H-bonding interaction with phenols, thereby facilitating thiol ionization at acidic pH. Stahl and Jencks showed that, compared with oxygen anions of similar pKa, thiol anions cause larger shifts in the infrared stretching frequency of hydrogen-bonded acids in non-aqueous solvents, a behavior consistent with a larger contribution of covalent interaction to hydrogen bonds of sulfur compared with oxygen anions [38]. Thapa and Schlegel included up to three explicit molecules of water hydrogen-bonded to the sulfur of thiols/thiolates in density functional theory calculations, which was found to lower the deviation between theoretical and experimental pKa values of thiols [39]. It should be remembered that small pH changes may lead to large increases in the oxidation rate of thiols; for example, Pryor et al. showed that cysteine may be oxidized to cysteine by NO in aqueous solution at pH ≥ 5, but not so at pH < 4 [40]. Even a small enhancement in thiol ionization can expedite the generation of thiyl radicals during oxidation, which is expected to strengthen the antioxidative ability of thiols [25,36].

In cases when phenols are oxidized faster than thiols (which is the case with the FRAP reagent, known to exhibit slow kinetics toward thiols), the Michael addition of nucleophilic thiols to quinones (i.e., emerging as the oxidation products of phenols) may rapidly produce new products (thiol-quinone adducts), depending on the nature, number, and position of substituents on the quinone, and this addition may also give rise to overoxidation of phenols and thiols via redox cycling involving molecular oxygen and ROS [41,42]. Overall, such interactions may play important parts to explain the seemingly high synergy experimentally observed for thiol-phenol mixtures using the FRAP method.

Cysteine can be oxidized to form several different products. These include the thiyl radical (–S^•^) by a one-electron transition, sulfenic acid (–SOH), and disulfide (–S–S–) by a two-electron transition, sulfinic acid (–SO_2_H) by a four-electron transition, and eventually sulfonic acid (–SO_3_H) by a six-electron transition [43]. Reversible enzymatic oxidation of GSH produces oxidized glutathione (GSSG), meaning that 2 GSH molecules lose two electrons to oxidize to GSSG [44]. The CUPRAC method assigns a TEAC coefficient of about 0.5 to either cysteine or GSH, which is in accordance with the reversible physiological oxidation of these biologically important thiols. On the other hand, overoxidation of thiols (such as irreversible 4-e and 6-e oxidations to sulfinic and sulfonic acids, respectively) represents physiologically irrelevant oxidations, and such reactions, if experimentally observed, do not correspond to a true “synergy” between antioxidants but instead—in the view of the authors—reflects an inherent error of the used TAC methods because they cannot effectively simulate physiological redox reactions in vitro. It is known that the TEAC values of either FRAP or ABTS methods assigned to cysteine and GSH are quite different from 0.5, which may change further in the analysis of mixtures [35].

The formation of stable Hg(II)-thiol complexes proved to be successful for independent determination of the polyphenolic content of thiol-phenol mixtures using all three TAC assay methods, because the strongly-complexed thiol components of the mentioned mixtures did not respond to these spectrophometric tests. Addition of Hg(II) to protein-phenol mixtures was basically unsuccessful in achieving additivity of TAC values, probably because of the fact that most protein thiols are not freely available for metal complexation and are buried within the macromolecular backbone, and that phenolic oxidation products (quinones) may bind site-specifically to proteins through thiol addition and addition-elimination reactions [45]. Thus, the absence or scarcity of free RSH groups in proteins rule out the possibility of additivity correction via RS-Hg^+^ complexation. To conclude with future recommendations, it can be stated that the CUPRAC method is capable of TAC determination of thiol-phenol mixtures in a perfectly additive manner without Hg(II) correction for thiol components.

## 4. Materials and Methods

### 4.1. Instrumentation and Chemicals

Chemicals and samples were weighed using a Radwag AS 220/C/2 analytical balance (Radwag, Radom, Poland); pH measurements were performed using a HANNA HI 221 pH-meter (Hanna Instruments, Woonsocket, RI, USA), and an Elmasonic ultrasonic water bath was used to dissolve chemicals. A Varian Cary 100 Bio UV-VIS spectrophotometer (Varian, Sydney, Australia) with matched HELLMA quartz cuvettes (light path = 10 mm) was used for optical absorption measurements.

All reagents were of analytical grade and purchased from the corresponding sources: iron(III) chloride hexahydrate, copper(II) chloride dihydrate, potassium dihydrogen phosphate, potassium monohydrogen phosphate, mercury(II) acetate, dodecyl sulfate sodium salt, tri-sodium citrate-5,5-hydrate, and bovine serum albumin were purchased from E. Merck (Darmstadt, Germany); *N*-acetyl-l-cysteine, 2,4,6-Tris(2-pyridyl)-*S*-triazine (TPTZ) were from Fluka (St. Louis, MO, USA); caffeic acid, (±)-catechinhydrate, epicatechin, dl-homocysteine, 2,2′-azinobis(3-ethylbenzothiazoline-6-sulfonic acid) diammonium salt (ABTS), neocuproine (2,9-dimethyl-1,10-phenantroline) hydrochloride, casein from bovine milk, trizma, and glycine were obtained from Sigma (Sigma Chemical Co., St. Louis, MO, USA); gallic acid, l-glutathione (reduced), acetic acid, ethanol, potassium persulfate, urea, and trichloroacetic acid (TCA) were purchased from Sigma-Aldrich (St. Louis, MO, USA); l-cysteine from Aldrich and sodium acetate trihydrate, hydrochloric acid, sodium hydroxide, sodium chloride, and ammonium acetate were from Riedel de Haen (Seelze, Germany).

### 4.2. Stock and Working Solutions

#### 4.2.1. Antioxidant (AOx) Solutions

Stock solutions of phenolic (GA, CFA, CAT, EC) and thiol-type antioxidants were prepared at 10 mM concentration. GSH, NAC were dissolved and diluted to volume with distilled water. HCYS CYS were dissolved with 0.5 mL of 1.0 M HCl and diluted to volume with distilled water. Solutions of phenolic AOxs were prepared in ethyl alcohol. For preparing working solutions of FRAP method, stock solutions of phenolic AOxs were diluted 100 times with ethanol and stock solutions of thiol-type AOxs were diluted 50 times with distilled water. For use in the ABTS method, AOx working solutions were diluted to the following concentrations: GA 4.0 × 10^−5^ M, CFA 2.0 × 10^−4^ M, CAT and EC 5.0 × 10^−5^ M, thiol-type antioxidants were at 1.0 × 10^−4^ M. For the CUPRAC method, working solutions of CAT, EC, CFA were at 1.0 × 10^−4^ M, GA was at 2.0 × 10^−4^ M and thiol-type AOxs at 1.0 × 10^−3^ M.

#### 4.2.2. Bovine Serum Albumin (BSA) Solution

To prepare a solution at 0.125% (*w*/*v*) for ABTS method and at 2.0% (*w*/*v*) for the CUPRAC method, BSA was dissolved with distilled water.

#### 4.2.3. Casein Solution

To obtain a solution at 0.5% (*w*/*v*) for ABTS/TEAC method and 1.0% (*w*/*v*) for the CUPRAC method, suitable amounts of casein were weighed, 0.4 mL of 1.0 M NaOH, distilled water was added, and the mixture was slightly heated. The pH of the solution was adjusted to 8.0 with 1.0 M HCl, and diluted to volume with distilled water.

#### 4.2.4. Protein Dissolution Buffer (pH 6.8)

For preparation of protein dissolution buffer, 50 mM tris (hydroxymethyl)aminomethane, 2% (*w*/*v*) sodium dodecyl sulfate and 8.0 M urea solutions were mixed, and the pH was adjusted to 6.8 with 6.0 M HCl.

#### 4.2.5. Egg White Protein Solution

Egg white and yolk were separated; egg white was suspended in distilled water using an ultrasonic water bath, and then filtered. Five milliliters of an aliquot was withdrawn, 5.0% TCA (*w*/*v*) was added to precipitate the proteins, and the mixture was centrifuged at 5000 rpm for 5 min. The separated precipitate was washed with distilled water 3–4 times and redissolved with protein dissolution buffer. Egg white protein solution was diluted 20 times with distilled water prior to use for the ABTS method, and it was used directly for the CUPRAC method.

#### 4.2.6. Mercury(II) Acetate Solution

A stock solution at 1.0 × 10^−2^ M concentration was prepared by dissolving a suitable amount of Hg(Ac)_2_ with 0.5 mL of 1.0 M HCl and distilled water. The working solutions at 5.0 × 10^−4^ M for use in the FRAP method and at 1.0 × 10^−3^ M for use in the ABTS method were prepared from the stock solution by suitable dilution with distilled water.

### 4.3. FRAP Method Reagents

Buffer solution at pH 3.6 (0.3 M): A weight of 3.1 g of sodium acetate was dissolved in distilled water, 16.0 mL acetic acid was added to the NaAc solution, and diluted to 1.0 L.

FeCl_3_·6H_2_O solution (2.0 × 10^−2^ M): A suitable amount of FeCl_3_·6H_2_O was dissolved using 0.5 mL of 1.0 M HCl and diluted to 25 mL with distilled water.

2,4,6-Tris(2-pyridyl)-S-triazine (TPTZ) solution: TPTZ solution at 1.0 × 10^−2^ M concentration was prepared by dissolving the suitable weight of TPTZ in ethyl alcohol.

FRAP reagent: pH 3.6 buffer solution:FeCl_3_·6H_2_O:TPTZ solutions were mixed at 10:1:1 volume ratio.

### 4.4. ABTS Method Reagents

ABTS/persulfate solution: 7.0 mM ABTS solution in distilled water was mixed with 2.45 mM potassium persulfate (K_2_S_2_O_8_) and let to stand for 12–16 h in the dark; then it was diluted 40 times with 5 mM phosphate buffered saline (PBS) solution at pH 7.4.

Phosphate-buffered saline (PBS) solution (pH 7.4): 100 mM KH_2_PO_4_ solution was added drop by drop onto 50 mL of 100 mM K_2_HPO_4_ to obtain a buffer solution at pH 7.4; 0.5 g NaCl was added and diluted to 5.0 mM with distilled water.

### 4.5. CUPRAC Method Reagents

CuCl_2_·2H_2_O solution: Prepared from CuCl_2_·2H_2_O in distilled water at 1.0 × 10^−2^ M concentration.

Neocuproine (Nc) solution: Prepared from neocuproine hydrochloride in ethyl alcohol at 7.5 × 10^−3^ M concentration.

Ammonium acetate (NH_4_Ac) solution: Prepared from NH_4_Ac in distilled water at 1.0 M concentration.

Urea buffer at pH 7: Tris (hydroxymethyl)aminomethane, glycine, sodium citrate, and urea at 0.086 M, 0.09 M, 4 mM, and 8.0 M final concentrations, respectively, were mixed and pH was adjusted to 7.0 with 6.0 M HCl.

Standard tris buffer at pH 8: Tris (hydroxymethyl)aminomethane, glycine, and sodium citrate at 0.086 M, 0.09 M, and 4 mM final concentrations, respectively, were mixed and pH was adjusted to 8.0 with 6.0 M HCl solution.

### 4.6. FRAP Method

Application of FRAP to a phenolic AOx and/or thiol compound: 0.1 mL thiol solution + x mL of phenolic antioxidant solution + (0.4 − x) mL ethyl alcohol + 0.1 mL distilled water (or 0.1 mL of 5.0 × 10^−4^ M Hg(Ac)_2_) and 3.0 mL of FRAP reagent solution were mixed respectively. The reaction mixture was let to stand at room temperature for six minutes and absorbance was read at 595 nm against reagent blank.

Optimization of the Hg(Ac)_2_ amount for FRAP Method: To obtain stable Hg-thiol complexes, Hg(II) salt was added to thiol + polyphenol mixtures. GA was used as phenolic and CYS as thiol type AOx, and a series of thiol:Hg(Ac)_2_ mol ratios were investigated. For this purpose, three types of solution were prepared, namely GA alone, GA + CYS and GA + CYS + Hg^2+^ mixtures. The amount of (GA + CYS) was kept constant while Hg^2+^ amount was varied. A series of CYS:Hg^2+^ solutions were prepared at 1:0.5, 1:1, 1:2.5, 1:5, and 1:10 mol ratios, and the FRAP method was applied.

FRAP method experiments: Different binary mixtures of thiols and phenolic AOx were tested with the FRAP method. To obtain binary mixtures, 0.1 mL of 2.0 × 10^−4^ M CYS, GSH, NAC, and HCYS were taken and different volumes of 1.0 × 10^−4^ M polyphenols were added to each thiol solution individually. For this purpose, different volumes ranging between 0.05–0.3 mL of GA, 0.1–0.4 mL of CFA, CAT, and EC were mixed and the FRAP method was applied. Each mentioned AOx compound (thiol or phenol) was tested individually and absorbance values were recorded. For binary mixtures to control additivity, the mathematical sum of these values were calculated and compared with experimental findings. Then all experiments were repeated in the presence of 0.1 mL of 5.0 × 10^−4^ M Hg(Ac)_2_. Since proteins precipitated in the presence of ethanol, protein mixtures could not be tested.

### 4.7. ABTS Method

Application of ABTS/TEAC to a phenolic AOx and/or thiol compound: 0.1 mL thiol solution + 0.4 mL distilled water, x mL phenolic antioxidant + (0.5 − x) mL ethanol + 2.0 mL ABTS^+^ were mixed in this order. After the reaction mixture was let to stand for six minutes at room temperature, absorbance was read against PBS at 734 nm.

Application of ABTS/TEAC to a protein-phenol antioxidant mixture: 1.0 mL PBS + 0.3 mL protein sample solution + 0.1 mL distilled water + x mL phenolic antioxidant + (0.5 − x) mL ethanol + (0.1 mL Hg(Ac)_2_) + 1.0 mL ABTS/persulfate (1:20 diluted with PBS) were mixed, and absorbance at 734 nm was read after 6 min standing.

Optimization of the amount of Hg(Ac)_2_ for the ABTS/TEAC Method: For 0.1 mL of 1.0 × 10^−4^ M CYS, Hg(II) salt was added at different mol ratios between 1:1–1:10 and ABTS/TEAC method was applied. The same amount of Hg(II) was also added to the reference (blank) solution. Both reference and CYS-Hg(II) samples were read against PBS, and the difference calculated (∆A = A_ref._ − A_sample_).

### 4.8. ABTS Tests for Mixtures of Polyphenolic and Thiolic AOx Compounds

To investigate the effect of thiols on the determination of phenolic AOx compounds, different binary and ternary mixtures were prepared. To prepare binary mixtures, 0.1 mL volumes were taken from 1.0 × 10^−4^ M GSH, CYS, NAC, HCYS, and each aliquot was mixed with 0.1–0.4 mL of 2.0 × 10^−4^ M CFA, 0.1–0.4 mL of 4.0 × 10^−5^ M GA and 0.05–0.25 mL of 5.0 × 10^−5^ M CAT and EC separately in the presence or absence of 0.1 mL of 1.0 × 10^−3^ M Hg^2+^. Using 0.15 mL CAT and 0.1 mL EC, CFA, CYS, and GSH; CYS + EC + GA; CYS + CFA + CAT; CYS + GA + CAT; GSH + EC + GA; GSH + CFA + CAT; GSH + GA + CAT; GSH + CFA + EC ternary mixtures were tested in the presence or absence of Hg(II) as stated earlier.

### 4.9. ABTS/TEAC Tests for Polyphenol–Protein Mixtures

To investigate polyphenol-protein interactions in the ABTS/TEAC method, three types of proteins were used: 0.125% BSA, 0.5% casein, and 1:20 diluted egg white protein solution. Different volumes between 0.1–0.4 mL of 2.0 × 10^−4^ M CFA were added to 0.2 mL of casein solution, and the ABTS/TEAC method was applied. Then, different volumes between 0.1–0.5 mL of 2.5 × 10^−5^ M CAT were added to 0.2 mL of 0.5% casein. These phenolic AOx compounds were also added to 0.1 mL of 0.125% BSA. The same antioxidant mixture was also prepared with 0.3 mL of 1:20 diluted egg white protein solution. Experiments were repeated in the presence and absence of 0.1 mL of 1.0 × 10^−3^ M Hg^2+^.

### 4.10. CUPRAC Method

Application of CUPRAC to a phenolic AOx and/or thiol compound: 1.0 mL CuCl_2_·2H_2_O + 1.0 mL Nc + 1.0 mL NH_4_Ac, x mL phenolic antioxidant + (1 − x) mL ethanol + 0.2 mL thiol solution, and 0.8 mL distilled water were mixed in this order. The mixture was let to stand for 30 min at room temperature. Absorbance was read at 450 nm against a reagent blank.

Application of CUPRAC to a protein-phenolic antioxidant mixture: 1.0 mL CuCl_2_·2H_2_O + 1.0 mL Nc, 2 mL pH 7 urea buffer + 1.0 mL standard tris pH 8.0 buffer + x mL phenolic antioxidant + (0.5 − x) mL ethanol + 0.4 mL protein and 0.1 mL distilled water were mixed, and the measurement was also made after 30 min standing. Here, NH_4_Ac buffer was replaced with urea buffer to prevent protein precipitation [34].

### 4.11. CUPRAC Measurements of Binary and Ternary Thiol-Phenol Mixtures

To prepare binary and ternary mixtures, 1.0 × 10^−3^ M thiol compounds (CYS, HCYS, NAC, GSH), 2.0 × 10^−4^ M GA, 1.0 × 10^−4^ M CAT, EC, and CFA were used. To prepare binary mixtures (i) 0.1–0.4 mL of GA; (ii) 0.2–0.6 mL of CFA; and (iii) 0.1–0.5 mL CAT (or EC) were added to 0.2 mL of thiol solution (CYS, HCYS, NAC, GSH) individually. For ternary mixtures, 0.2 mL aliquots of the corresponding components of mixtures CYS + EC + GA; CYS + CFA + CAT; CYS + GA + CAT; CYS + CFA + EC were mixed; then, the same phenolic AOx compounds were mixed with 0.2 mL of GSH instead of CYS.

### 4.12. CUPRAC Measurements for Protein–Phenol Mixtures

Aliquots of 0.4 mL of 1.0% casein, 2.0% BSA, and egg white protein solution (without any dilution) were mixed with 0.1–0.5 mL of 1.0 × 10^−4^ M CFA, 0.1–0.5 mL of 1.0 × 10^−4^ M CAT, EC, and 0.1–0.4 mL of 2.0 × 10^−4^ M GA individually. The effect of Hg^+^ was also investigated for the CUPRAC method, and in the presence of Hg(II), the results were not significantly different.

## 5. Conclusions

Accurate and precise measurement of the antioxidant capacity of thiol-phenol mixtures is a priority challenge in food analysis since any synergistic or antagonistic interactions between the mixture components may affect the shelf-life of food. Synergism between antioxidants is defined as the more effective behavior of mixtures compared to single compounds, and usually used in the sense of protection of one antioxidant by the other against lipid peroxidation. However, in non-competitive spectrophotometric TAC assays, a chromogenic redox probe oxidizes the tested antioxidants and changes color, where the additivity of TAC values is a consequence of Beer’s law applied to mixtures. Three of the most common spectrophotometric TAC determination methods (FRAP, ABTS/TEAC, and CUPRAC) were applied to different thiol-polyphenol and protein-polyphenol mixtures, where the most additive results were obtained for the CUPRAC method; the largest deviations were seen for the FRAP method, whereas ABTS/TEAC exhibited medium level deviations. Thus, overoxidation of thiol-phenol mixtures primarily observed in the FRAP method (and to a minor extent in the ABTS method) seems to arise from systematic methodological errors. The best way to correct this error (i.e., deviations from additivity) was to add Hg(II) salts to strongly complex the thiol components so that the true TAC values manifested by phenols in such mixtures could be observed. Applying the same measure (i.e., Hg(II) complexation) to protein-phenol mixtures was not as effective, possibly due to the fact that most protein thiols are buried within the macromolecular backbone. The CUPRAC method worths to be recommended for the TAC determination of thiol-phenol mixtures to avoid systematic errors and get additive results.

## Figures and Tables

**Table 1:** Thiol:Hg^2+^ mol ratio optimization for ferric reducing antioxidant power (FRAP) method.

Sample	A_(FRAP)_^1^
Only GA	0.3130 ± 0.016
Only CYS	0.0187 ± 0.019
GA + CYS	0.6455 ± 0.023
GA + (1:0.5) CYS:Hg	0.3865 ± 0.029
GA + (1:1.0) CYS:Hg	0.3149 ± 0.011
GA + (1:2.5) CYS:Hg	0.3226 ± 0.006
GA + (1:5.0) CYS:Hg	0.3656 ± 0.016

^1^ Absorbance = $\bar{x}$ ± (t_0.95_s/N^½^); *N* = 5 ($\bar{x}$ = mean, s = standard deviation). GA, gallic acid; CYS, cysteine.

**Table 2:** FRAP method results for individual thiols, phenolic antioxidant (AOx) solutions, and binary mixtures. In the experiments, 0.1 mL of 2.0 × 10^−4^ M thiols and different volumes of 1.0 × 10^−4^ M phenolic AOx compounds were used in the presence and absence of 5.0 × 10^−4^ M Hg^2+^.

Volume (V) (mL) of Mixture Components (AOx and/or Thiol)	A_exp_^1^ (in the Absence of Hg^2+^)	A_exp_^1^ (in the Presence of Hg^2+^)	Relative Error % (in the Absence of Hg^2+^)	Relative Error % (in the Presence of Hg^2+^)
0.10 mL HCYS	0.0016 ± 0.002	-	-	-
0.10 mL NAC	0.1430 ± 0.010			
0.05 mL GA	0.1557 ± 0.001	-	-	-
0.10 mL GA	0.2867 ± 0.016			
0.20 mL GA	0.6060 ±0.008			
0.30 mL GA	0.9551 ± 0.005			
0.05 mL GA + 0.1 mL HCYS	0.3865 ± 0.024	0.1695 ± 0.003	148.2	8.9
0.10 mL GA + 0.1 mL HCYS	0.5307 ± 0.034	0.3039 ± 0.013	85.1	6.0
0.20 mL GA + 0.1 mL HCYS	1.0427 ± 0.025	0.6314 ± 0.009	72.1	4.2
0.30 mL GA + 0.1 mL HCYS	1.3319 ± 0.012	0.9636 ± 0.007	39.4	0.9
0.05 mL GA + 0.1 mL NAC	0.5217 ± 0.012	0.1580 ± 0.007	74.7	1.5
0.10 mL GA + 0.1 mL NAC	0.7309 ± 0.032	0.3092 ± 0.021	70.1	7.8
0.20 mL GA + 0.1 mL NAC	1.0345 ± 0.014	0.6636 ± 0.009	38.1	9.5
0.30 mL GA + 0.1 mL NAC	1.3758 ± 0.039	0.9709 ± 0.021	25.3	1.6

^1^ Absorbance = $\bar{x}$ ± (t_0.95_s/N^½^); *N* = 5 ($\bar{x}$ = mean, s = standard deviation). HCYS: homocysteine; NAC: *N*-acetyl-l-cysteine.

**Table 3:** Optimization of thiol:Hg^2+^ mol ratio for the ABTS/TEAC method.

Sample	A_(ABTS/TEAC)_
Ref. (without Hg)	0.9306
Ref. (1:1 Hg)	0.9382
Ref. (1:5 Hg)	0.9277
Ref. (1:10 Hg)	0.9316
Only CYS	0.6967
1:1 CYS:Hg(II)	0.8396
1:5 CYS:Hg(II)	0.9146
1:10 CYS:Hg(II)	0.9162

ABTS/TEAC: 2,2′-azinobis(3-ethylbenzothiazoline-6-sulfonic acid) diammonium salt/trolox equivalent antioxidant capacity; [30].

**Table 4:** The absorbance drops (∆A) using the ABTS/TEAC method for individual components or binary mixtures consisting of 0.1 mL of 1.0 × 10^−4^ M HCYS (or NAC) and 0.05–0.25 mL of 5.0 × 10^−5^ M catechin (CAT).

V (mL) of AOx (Thiol, Catechin, or Mixture)	∆A_exp._^1^ (without Hg^2+^)	∆A_exp._^1^ (with Hg^2+^)	RE% (without Hg^2+^)	RE% (with Hg^2+^)
0.10 mL HCYS	0.2011 ± 0.026	-	-	-
0.10 mL NAC	0.2825 ± 0.034	-	-	-
0.05 mL CAT	0.0738 ± 0.013	0.0482 ± 0.026	-	-
0.10 mL CAT	0.1243 ±0.019	0.1134 ± 0.018	-	-
0.15 mL CAT	0.2099 ± 0.024	0.1791 ± 0.008	-	-
0.20 mL CAT	0.2658 ± 0.015	0.2912 ± 0.013	-	-
0.25 mL CAT	0.3856 ± 0.008	0.3970 ± 0.028	-	-
HCYS + 0.05 mL CAT	0.2958 ± 0.015	0.0530 ± 0.019	7.6	5.4
HCYS + 0.10 mL CAT	0.3864 ± 0.015	0.1216 ± 0.027	18.8	5.2
HCYS + 0.15 mL CAT	0.4528 ± 0.013	0.1939 ± 0.021	10.2	7.0
HCYS + 0.20 mL CAT	0.5434 ± 0.018	0.3004 ± 0.037	16.4	2.4
HCYS + 0.25 mL CAT	0.6475 ± 0.023	0.4027 ± 0.021	10.4	1.1
NAC + 0.05 mL CAT	0.3110 ± 0.015	0.0754 ± 0.034	12.7	6.6
NAC + 0.10 mL CAT	0.3528 ± 0.010	0.1430 ± 0.010	13.3	8.5
NAC + 0.15 mL CAT	0.3889 ± 0.027	0.1898 ± 0.021	21.0	0.2
NAC + 0.20 mL CAT	0.4693 ± 0.009	0.2553 ± 0.022	14.4	10.0
NAC + 0.25 mL CAT	0.5227 ± 0.047	0.3363 ± 0.023	21.8	6.6

^1^ ∆A = $\bar{x}$ ± (t_0.95_s/N^½^); *N* = 5 ($\bar{x}$ = mean, s = standard deviation).

**Table 5:** Cupric Ion Reducing Antioxidant Capacity (CUPRAC) absorbances of ternary mixtures of 0.2 mL volumes of 1.0 × 10^−3^ M thiol compounds (CYS, HCYS, NAC, GSH) with polyphenols, namely 2.0 × 10^−4^ M GA and 1.0 × 10^−4^ M CAT, EC, and CFA.

Sample	A_exp._^1^	A_theo_	RE%
CYS	0.3061 ± 0.011	-	-
GSH	0.3222 ± 0.008	-	-
CAT	0.2274 ± 0.018	-	-
EC	0.2826 ± 0.016	-	-
CFA	0.1924 ± 0.024	-	-
GA	0.3988 ± 0.030	-	-
CYS + EC + GA	0.9930 ± 0.037	0.9875	0.6
CYS + CFA + CAT	0.7468 ± 0.028	0.7259	2.9
CYS + GA + CAT	0.9548 ± 0.038	0.9323	2.4
CYS + CFA + EC	0.7848 ± 0.028	0.7811	0.5
GSH + EC + GA	1.0131 ± 0.021	1.0036	1.0
GSH + CFA + CAT	0.7690 ± 0.021	0.7420	3.5
GSH + GA + CAT	0.9872 ± 0.027	0.9484	4.1
GSH + CFA + EC	0.8324 ± 0.019	0.7972	4.4

^1^ Absorbance= $\bar{x}$ ± (t_0.95_s/N^½^); *N* = 5 ($\bar{x}$ = mean, s = standard deviation). GSH: glutathione; EC: epicatechin; CFA: caffeic acid.

## Abbreviations

ET	Electron transfer
TAC	Total antioxidant capacity
ROS/RNS	Reactive oxygen/nitrogen species
AOx	Antioxidant
HSA	Human serum albumin
Hb	Hemoglobin
TEAC	Trolox equivalent antioxidant capacity
GSH	Glutathione
FRAP	Ferric reducing antioxidant power
ORAC	Oxygen Radical Absorbance Capacity
CUPRAC	Cupric ion reducing antioxidant capacity
ABTS	2,2′-azinobis(3-ethylbenzothiazoline-6-sulfonic acid) diammonium salt
DPPH	2,2-diphenyl-1-picryl-hydrazyl
HSAB	Hard and Soft Acids and Bases
GA	Gallic acid
CYS	Cysteine
HCYS	Homocysteine
NAC	*N*-acetyl-l-cysteine
CFA	Caffeic acid
CAT	Catechin
EC	Epicatechin
BSA	Bovine serum albumin
RS	Thiyl radical
TPTZ	2,4,6-Tris(2-pyridyl)-*S*-triazine
PBS	Phosphate buffered saline
Nc	Neocuproine
